# Hepatic Mycotic Aneurysms in TAVI-Associated Infective Endocarditis Caused by *Streptococcus gallolyticus*: A Case Report

**DOI:** 10.3390/reports9040327

**Published:** 2026-09-27

**Authors:** Mihai Grigore, Camelia Nicolae, Horațiu Moldovan, Ana-Maria Balahura, Adriana-Mihaela Ilieșiu

**Affiliations:** 1 Cardio-Thoracic Department, Carol Davila University of Medicine and Pharmacy, 021021 Bucharest, Romania; mihai.grigore@umfcd.ro (M.G.); ana-maria.balahura@umfcd.ro (A.-M.B.);; 2 Internal Medicine and Cardiology Department, “Prof. Th. Burghele” Clinical Hospital, 050653 Bucharest, Romania

**Keywords:** TAVI-associated infective endocarditis, *Streptococcus gallolyticus*, hepatic artery mycotic aneurysm, prosthetic valve endocarditis, septic embolization, transcatheter aortic valve implantation, visceral artery aneurysm

## Abstract

**Background and Clinical Significance:** Infective endocarditis following transcatheter aortic valve implantation (TAVI) is a severe complication that may be associated with prosthetic valve dysfunction, local infectious extension, and systemic embolization. Visceral mycotic aneurysms are uncommon, particularly those involving the hepatic arterial circulation. **Case Presentation:** We report the case of an 80-year-old woman who developed *Streptococcus gallolyticus* TAVI-associated prosthetic valve endocarditis (TAVI-PVE) four months after valve implantation. Despite prolonged antimicrobial therapy and subsequent negative blood cultures, the clinical course was complicated by persistent prosthetic and periprosthetic echocardiographic abnormalities, cerebral and splenic embolic events, and new-onset abdominal pain. Contrast-enhanced computed tomography revealed two hepatic arterial aneurysmal lesions with surrounding inflammatory changes, consistent with mycotic aneurysms. After initially declining surgery, the patient underwent redo surgical aortic valve replacement; cultures of the surgically excised specimens were negative. Abdominal pain resolved and inflammatory markers normalized following surgery and continued antimicrobial therapy. Follow-up contrast-enhanced CT demonstrated marked interval enlargement of the hepatic arterial aneurysmal lesion to 28 mm × 28 mm × 26 mm despite clinical improvement and normalization of inflammatory markers. **Conclusions:** This case highlights that microbiological, clinical, and biological improvement does not necessarily indicate resolution of local cardiac or extracardiac structural complications and emphasizes the importance of repeated imaging, guideline-based antimicrobial therapy, timely source control, and multidisciplinary vascular management in complicated TAVI-PVE.

## 1. Introduction and Clinical Significance

Infective endocarditis remains a severe complication after valve replacement, particularly when prosthetic material is involved. With the increasing use of transcatheter aortic valve implantation (TAVI), TAVI-associated prosthetic valve endocarditis (TAVI-PVE) has become an important clinical concern, particularly in elderly and frail patients. Although its cardiac and systemic complications are increasingly recognized, visceral arterial involvement remains uncommon [1].

Mycotic aneurysms are rare but potentially life-threatening complications of infective endocarditis, generally resulting from septic embolization followed by infection and destruction of the arterial wall [2]. They occur in approximately 2% of patients with infective endocarditis and may involve intracranial or extracranial arterial territories [3]. Visceral involvement is considerably less frequent, with hepatic artery mycotic aneurysms representing a particularly uncommon manifestation [4,5,6].

Their clinical presentation may be nonspecific, ranging from incidental detection to abdominal pain, gastrointestinal bleeding, hemobilia, or rupture [6,7,8]. *Streptococcus gallolyticus*, although well recognized as a cause of infective endocarditis and for its association with gastrointestinal pathology, has only rarely been associated with extracranial mycotic aneurysms [9].

We report a rare case of *S. gallolyticus* TAVI-PVE complicated by persistent prosthetic valve abnormalities, systemic embolization, and hepatic artery mycotic aneurysms. The case emphasizes the importance of investigating new abdominal symptoms, distinguishing microbiological clearance from resolution of structural complications, reassessing surgical source control when complicated prosthetic valve disease persists, and continuing vascular surveillance despite apparent clinical improvement.

## 2. Case Presentation

An 80-year-old woman with arterial hypertension, dyslipidemia, atrial fibrillation on chronic oral anticoagulation, and heart failure with preserved left ventricular ejection fraction underwent transfemoral TAVI in September 2025 with implantation of an Edwards SAPIEN 3 Ultra 26 mm bioprosthesis (Edwards Lifesciences Corporation, Irvine, CA, USA) for severe degenerative aortic stenosis. The immediate post-procedural course was uneventful.

In January 2026, approximately four months after TAVI, she was admitted to another hospital with progressive dyspnea evolving over approximately two weeks to symptoms at rest, associated with orthopnea, marked fatigue, and a syncopal episode. She was hemodynamically stable but had significant weight loss and clinical features of chronic illness.

Laboratory evaluation demonstrated severe hypochromic microcytic anemia (Hb 7.2 g/dL), leukocytosis with neutrophilia, markedly increased inflammatory markers (C reactive-protein 178 mg/L), and elevated NT-proBNP (22.672 pg/mL). Blood cultures obtained during the initial admission were positive for *Streptococcus gallolyticus*, reported as resistant to penicillin and susceptible to cephalosporins.

The exact number and timing of the initially positive culture sets and the minimum inhibitory concentrations could not be reliably reconstructed from the available retrospective records. Importantly, repeat blood cultures obtained during the March 2026 re-admission were negative.

Transthoracic echocardiography raised suspicion of prosthetic valve dysfunction. Transesophageal echocardiography confirmed TAVI-PVE, revealing multiple mobile vegetations measuring up to approximately 10 mm attached to the transcatheter aortic valve prosthesis. Integrated echocardiographic assessment demonstrated severe prosthetic valve obstruction, with a peak transprosthetic velocity of 4.8 m/s, a mean gradient of 41 mmHg, and an indexed effective orifice area of 0.45 cm^2^/m^2^, together with mild intraprosthetic aortic regurgitation (EROA 7 mm^2^; vena contracta width 2 mm).

Empirical intravenous antimicrobial therapy was initiated with ceftriaxone and vancomycin. After microbiological identification of *S. gallolyticus*, ceftriaxone-based intravenous treatment was continued, with an overall intravenous treatment duration exceeding six weeks. The initial ceftriaxone–vancomycin combination was administered for approximately 14 days.

During hospitalization at another institution, the patient declined the recommended cardiac surgical intervention and refused continued inpatient treatment, requesting discharge. Antimicrobial therapy was subsequently changed to oral moxifloxacin 400 mg once daily plus rifampicin 600 mg once daily to allow continuation of treatment after discharge.

During the initial hospitalization, the patient developed transient dysarthria and hemiparesis, clinically interpreted as a probable cardioembolic transient ischemic event. This neurological complication preceded definitive cardiac surgery by 8 weeks. No intracranial hemorrhagic complication was documented in the available clinical records. Imaging also demonstrated splenic lesions consistent with embolic infarctions.

Given the established association between *S. gallolyticus* infection and gastrointestinal pathology, colonoscopy was performed and revealed a large pedunculated sigmoid polyp. Immediate removal was deferred because of anticoagulant treatment and the active cardiac condition. Following subsequent stabilization, the polyp was resected, and histopathological examination demonstrated tubular adenoma with low-grade dysplasia, completely resected.

Despite significant prosthetic dysfunction and systemic embolic complications providing a strong indication for surgery, the patient initially declined surgical treatment after repeated multidisciplinary discussions. Conservative management was therefore continued.

In March 2026, the patient was admitted in our hospital because of persistent dyspnea at rest and vertigo and, most importantly, new-onset abdominal pain localized to the epigastrium with radiation toward the left hypochondrium. Inflammatory markers remained elevated despite prolonged antimicrobial treatment (C-reactive protein–98 mg/L). Repeat blood cultures were negative.

Repeat transesophageal echocardiography demonstrated residual vegetative material measuring up to 4 mm, moderate intraprosthetic aortic regurgitation, mild paravalvular regurgitation, and moderate prosthetic obstruction. The transprosthetic peak velocity was 3.1 m/s and the indexed effective orifice area: 0.7 cm^2^/m^2^). Moderate intraprosthetic regurgitation was supported by a vena contracta width of 5 mm. A heterogeneous echogenic periprosthetic infiltrative formation was also identified, raising suspicion of persistent local/periprosthetic involvement (Figure 1).

The apparent change from severe to moderate prosthetic stenosis between examinations should not be interpreted as confirmed structural improvement. The examinations were obtained at different stages of the disease and under different hemodynamic conditions, and complete standardized serial quantitative Doppler datasets were not available for retrospective comparison.

Contrast-enhanced abdominal CT demonstrated two aneurysmal lesions involving the hepatic arterial circulation: a 0.6 cm saccular aneurysmal lesion of the common hepatic artery and a 0.4 cm pseudoaneurysmal dilatation of the right hepatic artery. Marked perivascular inflammatory changes were present, without active contrast extravasation or radiological evidence of rupture. Segmental thrombosis of the right hepatic artery with distal repermeabilization was also observed. Multiple splenic hypodense lesions compatible with previous embolic infarctions remained visible (Figure 2).

The saccular/pseudoaneurysmal morphology, surrounding inflammatory changes, and development of these lesions in the context of recently treated TAVI-PVE and previous systemic embolization strongly supported an infective origin.

No immediate endovascular or open vascular procedure was performed during this phase. In the absence of active bleeding or rupture and in the presence of unresolved prosthetic/periprosthetic structural disease requiring definitive cardiac source control, the immediate therapeutic priority was management of the cardiac prosthetic infection together with antimicrobial therapy and subsequent vascular surveillance.

During the March admission, intravenous ceftriaxone was reintroduced together with rifampicin. Despite negative repeat blood cultures, the coexistence of persistent prosthetic abnormalities, significant prosthetic dysfunction, suspected periprosthetic extension, and progressive extracardiac vascular complications indicated that microbiological clearance had not been accompanied by adequate anatomical source control.

Following renewed multidisciplinary discussion, the patient accepted surgical management and underwent redo surgical aortic valve replacement. Through a transverse aortotomy, the SAPIEN 3 Ultra transcatheter prosthesis was circumferentially dissected from the native aortic wall. The metallic frame was radially collapsed and the prosthesis was explanted en bloc. Intraoperative inspection demonstrated localized inflammatory and destructive involvement at the level of the aortic annulus, without extensive aortic-root destruction or involvement of the mitral–aortic continuity. Radical debridement of the affected tissue was performed. The remaining annular tissue was suitable for direct valve implantation; therefore, annular or aortic-root reconstruction was not required. A 23 mm Hancock surgical bioprosthesis (Medtronic plc, Galway, Ireland) was subsequently implanted in the debrided native annulus (Figure 3).

The explanted prosthesis and excised periprosthetic tissue were submitted for microbiological culture, which yielded no microbial growth in the context of prolonged preceding antimicrobial therapy. Repeat blood cultures were also negative.

The postoperative course was favorable from both hemodynamic and infectious perspectives but was complicated by complete atrioventricular block requiring implantation of a permanent dual-chamber pacemaker. According to the surgical assessment, the conduction disturbance was attributed to injury of the conduction tissue during dissection of the metallic transcatheter-valve frame from the interventricular septal region.

Postoperative echocardiography demonstrated a normally functioning surgical aortic bioprosthesis without significant paravalvular regurgitation. Transesophageal color Doppler imaging identified a small communication between the left ventricular outflow tract (LVOT) and the left atrium (Figure 4). This communication had not been identified on the available preoperative TEE examinations and was not observed intraoperatively. It was considered hemodynamically insignificant, with no clinical or echocardiographic evidence of significant hemodynamic compromise.

The exact mechanism of the LVOT–left atrial communication cannot be established with certainty. Given the periprosthetic infiltrative abnormality already documented on preoperative TEE and the subsequent radical surgical debridement, the communication may reflect infective periannular tissue weakening that became apparent or was extended during surgical manipulation and debridement, rather than an exclusively infectious or iatrogenic complication.

Following surgery, abdominal pain completely resolved, no further clinically apparent embolic events occurred, and serial laboratory assessments demonstrated normalization of inflammatory markers. The patient was subsequently discharged in stable clinical condition under multidisciplinary follow-up.

Importantly, subsequent contrast-enhanced CT performed in May 2026 demonstrated unfavorable anatomical evolution despite the favorable clinical and inflammatory response. Compared with the initial CT from March 2026, a single aneurysmal formation was identified at the site of the two previously described hepatic arterial lesions, showing marked interval enlargement and measuring 28 mm× 28 mm× 26 mm (Figure 2). The lesion demonstrated slow, progressive contrast opacification and was surrounded by a well-defined mural/perianeurysmal inflammatory cuff. The common hepatic artery entered the aneurysmal formation and subsequently lost its separate delineation. Distally, the gastroduodenal artery remained filiform, while the right and left hepatic arteries showed distal reperfusion through collateral arterial pathways. No additional aneurysmal lesions or new visceral ischemic changes were identified.

Thus, despite clinical improvement and normalization of inflammatory markers following cardiac source control and antimicrobial therapy, the hepatic arterial vascular complication demonstrated significant anatomical progression. Although currently asymptomatic, the patient was referred for specialized vascular/interventional evaluation to determine the optimal definitive treatment strategy.

The timeline summarizes the evolution from TAVI to *S. gallolyticus* TAVI-PVE in January 2026, prolonged antimicrobial therapy and systemic embolic complications, March 2026 re-admission with persistent structural disease and diagnosis of two hepatic arterial aneurysmal lesions, redo surgical aortic valve replacement, subsequent clinical and inflammatory improvement, and follow-up CT demonstrating marked anatomical progression to a single enlarged hepatic arterial aneurysmal formation, prompting specialized vascular/interventional evaluation (Figure 5).

## 3. Discussion

This case combines several unusual and clinically important features: TAVI-PVE caused by *Streptococcus gallolyticus*, persistent prosthetic and periprosthetic structural abnormalities despite prolonged antimicrobial therapy and microbiological clearance, systemic embolic complications, and hepatic artery mycotic aneurysms with subsequent anatomical progression despite clinical improvement.

Mycotic aneurysms are uncommon complications of infective endocarditis. In the national GAMES cohort, they occurred in approximately 2% of patients, with extracranial lesions representing only a proportion of this already uncommon complication [3]. Hepatic arterial involvement is particularly rare. Boukobza et al. identified hepatic artery aneurysms in only 10 of 623 patients with infective endocarditis and emphasized that these lesions may be clinically silent and detected only through systematic or symptom-driven imaging [6].

The temporal course in our patient illustrates this diagnostic challenge. Cerebral and splenic embolic manifestations preceded recognition of hepatic arterial involvement. The hepatic vascular lesions became clinically apparent only when persistent inflammatory activity was accompanied by new abdominal pain, prompting contrast-enhanced abdominal CT.

The microbiological context is also noteworthy. *S. gallolyticus* is strongly associated with gastrointestinal pathology, and colonoscopy in our patient demonstrated a large sigmoid polyp. Subsequent resection showed benign histopathology. Although the association between *S. gallolyticus* and infective endocarditis is well established, extracranial mycotic aneurysms caused by this microorganism are rarely reported [9,10]. Spring et al. described multifocal extracranial mycotic aneurysms in a patient with *S. gallolyticus* endocarditis, including hepatic arterial involvement [9].

Several features strongly supported an infective origin of the hepatic arterial lesions: recently documented *S. gallolyticus* TAVI-PVE with mobile vegetations, embolic involvement of multiple vascular territories, saccular/pseudoaneurysmal morphology, and marked surrounding inflammatory changes. These findings are consistent with septic embolic seeding and secondary arterial wall destruction [2].

Importantly, blood cultures obtained during the March re-admission were negative, and cultures of the surgically excised specimens also yielded no microbial growth. These findings were obtained after prolonged antimicrobial exposure and therefore indicate the absence of documented ongoing bacteremia rather than resolution of the structural consequences of infection. Residual vegetative material, prosthetic dysfunction, suspected periprosthetic involvement, and hepatic arterial vascular complications remained present despite negative cultures.

A similar distinction became evident during postoperative follow-up. Redo surgical aortic valve replacement and continued antimicrobial therapy were followed by resolution of abdominal pain and normalization of inflammatory markers. Nevertheless, follow-up contrast-enhanced CT in May 2026 demonstrated unfavorable anatomical evolution. At the site of the two previously described hepatic arterial lesions, a single aneurysmal formation was identified, showing marked interval enlargement and measuring 28 mm × 28 mm × 26 mm. The lesion demonstrated slow, progressive contrast opacification and remained surrounded by a well-defined mural/perianeurysmal inflammatory cuff. The common hepatic artery entered the aneurysmal formation and subsequently lost its separate delineation, while the right and left hepatic arteries showed distal reperfusion through collateral arterial pathways.

Thus, microbiological, clinical, and biological improvement cannot be considered evidence of anatomical vascular resolution. In our patient, the hepatic arterial complication progressed despite apparent control of the underlying cardiac infection, directly emphasizing the importance of serial vascular imaging after diagnosis of a mycotic aneurysm.

Serial vascular imaging is essential because mycotic aneurysms may persist, regress, enlarge, thrombose, or rupture despite apparent control of the underlying infection [4,5,6]. The prognosis of hepatic mycotic aneurysms is influenced by rupture risk, anatomical location, lesion morphology and progression, persistent infection, and the feasibility of definitive vascular treatment. Rupture may result in life-threatening intraperitoneal hemorrhage, gastrointestinal bleeding, or hemobilia. Persistent or enlarging lesions therefore require structured imaging follow-up even when the underlying infective endocarditis appears clinically and microbiologically controlled.

Management should be individualized according to symptoms, morphology, dimensions and interval growth, anatomical location, rupture status, hepatic perfusion, comorbidity burden, and local expertise [4,5]. Endovascular treatment is increasingly favored when anatomically feasible, particularly in high-risk patients, whereas open vascular surgery may be required in selected cases with unfavorable anatomy or when an endovascular approach cannot adequately exclude the lesion while preserving hepatic perfusion.

Boukobza et al. reported that some small hepatic artery aneurysms associated with infective endocarditis may regress during antimicrobial treatment, whereas endovascular embolization represents an important therapeutic option for selected lesions [6]. In contrast, our patient demonstrated marked interval enlargement of the hepatic arterial aneurysmal lesion despite resolution of abdominal symptoms and normalization of inflammatory markers. The lesion therefore cannot be regarded as a clinically irrelevant residual imaging finding.

Although currently asymptomatic, the patient has been referred for specialized vascular/interventional evaluation because of the marked anatomical progression. Definitive endovascular treatment will be considered according to the relationship of the lesion to the hepatic arterial branches, collateral circulation, and the ability to preserve adequate hepatic perfusion.

The antimicrobial strategy warrants particular attention. The transition to oral moxifloxacin plus rifampicin was an individualized, non-standard strategy adopted during hospitalization at another institution after the patient declined the recommended cardiac surgical intervention and refused continued inpatient treatment, requesting discharge. It should therefore not be interpreted as standard guideline-directed oral treatment for *S. gallolyticus* PVE.

This distinction is important because partial oral antimicrobial treatment is appropriate only in carefully selected clinically stable patients after an adequate intravenous treatment phase and after exclusion of uncontrolled local complications [1]. Our patient had significant prosthetic dysfunction, previous embolic complications, and persistent echocardiographic abnormalities and therefore did not meet the profile of an uncomplicated stable patient appropriate for routine oral step-down therapy.

The postoperative LVOT–left atrial communication also requires cautious interpretation. It was identified after redo surgery, had not been identified on the available preoperative TEE examinations, and was not observed intraoperatively. Nevertheless, this temporal association does not establish an exclusively iatrogenic origin.

Periannular extension of infective endocarditis may result in abscesses, pseudoaneurysms, and fistulous communications, whereas cardiac surgery and radical debridement may create, extend, or unmask abnormal communications. Romiti et al. described a pseudoaneurysm of the mitral–aortic intervalvular fibrosa and highlighted the potential relationship between infectious and postoperative mechanisms [11,12]. Accordingly, the LVOT–left atrial communication in our patient may reflect pre-existing infective periannular tissue weakening that became apparent or was extended during surgical manipulation and debridement, rather than an exclusively infectious or iatrogenic complication.

Several limitations should be acknowledged. The exact number and timing of the initially positive blood culture sets and minimum inhibitory concentrations could not be reconstructed retrospectively. Nevertheless, repeat blood cultures during the March re-admission and microbiological cultures of surgically excised specimens were documented as negative.

Despite these limitations, several practical messages emerge. New-onset abdominal pain in infective endocarditis, particularly after previous systemic embolization, should prompt investigation for visceral vascular complications. In the present case, follow-up CT demonstrated marked anatomical progression of the hepatic arterial lesion despite an asymptomatic clinical status and normalization of inflammatory markers. Finally, early TAVI-PVE with significant prosthetic dysfunction, embolization, or local extension warrants repeated multidisciplinary reassessment for definitive source control.

## 4. Conclusions

Hepatic artery mycotic aneurysms are rare but potentially life-threatening complications of infective endocarditis. This case highlights that microbiological and clinical improvement after antimicrobial therapy and cardiac source control does not necessarily correspond to resolution of extracardiac vascular complications. Despite a favorable clinical course, follow-up imaging demonstrated marked anatomical progression of the hepatic arterial aneurysmal lesion. Therefore, serial vascular imaging and multidisciplinary assessment, including Interventional Radiology evaluation when appropriate, remain essential in the management of these patients.

## Figures and Tables

**Figure 1:**
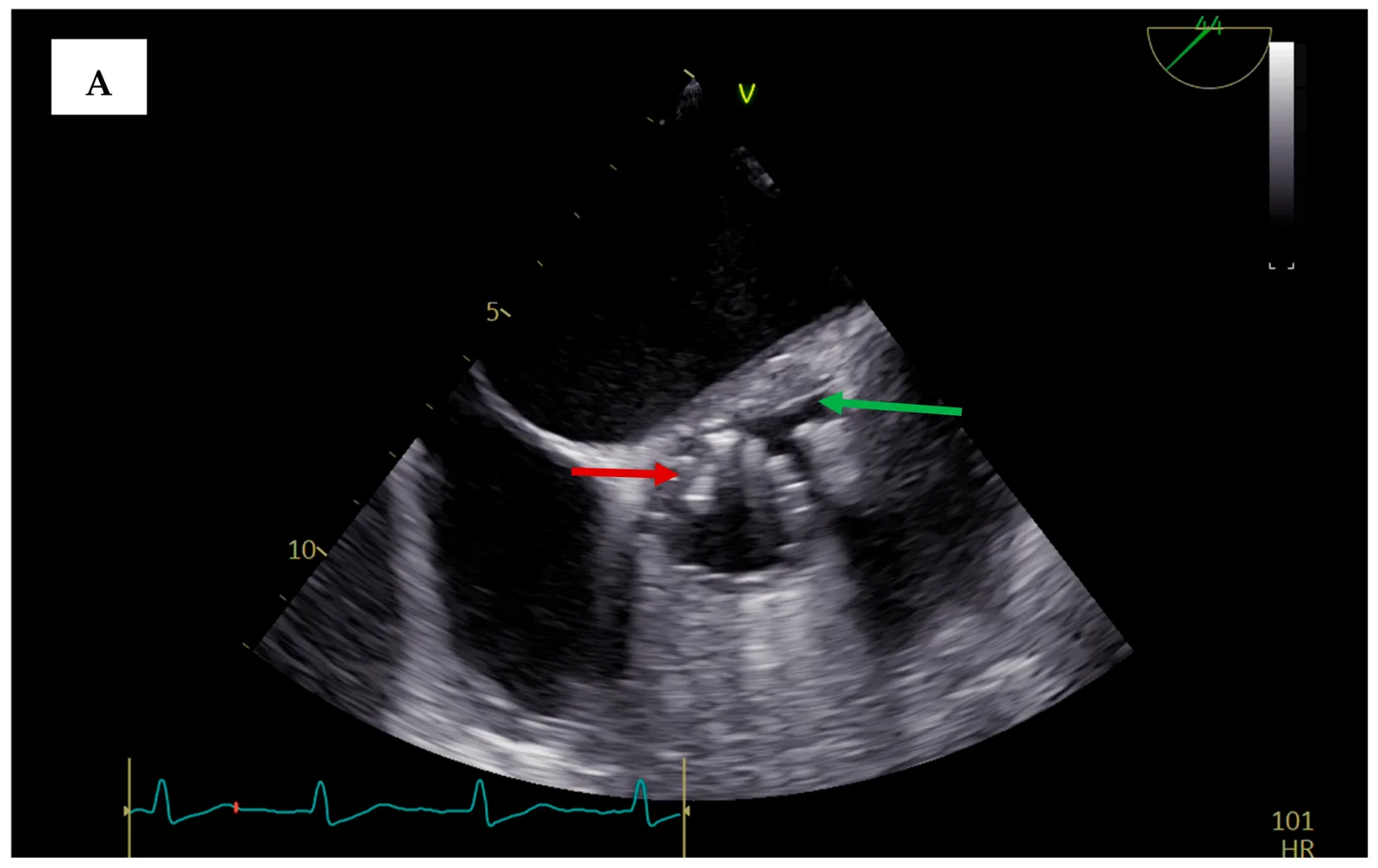

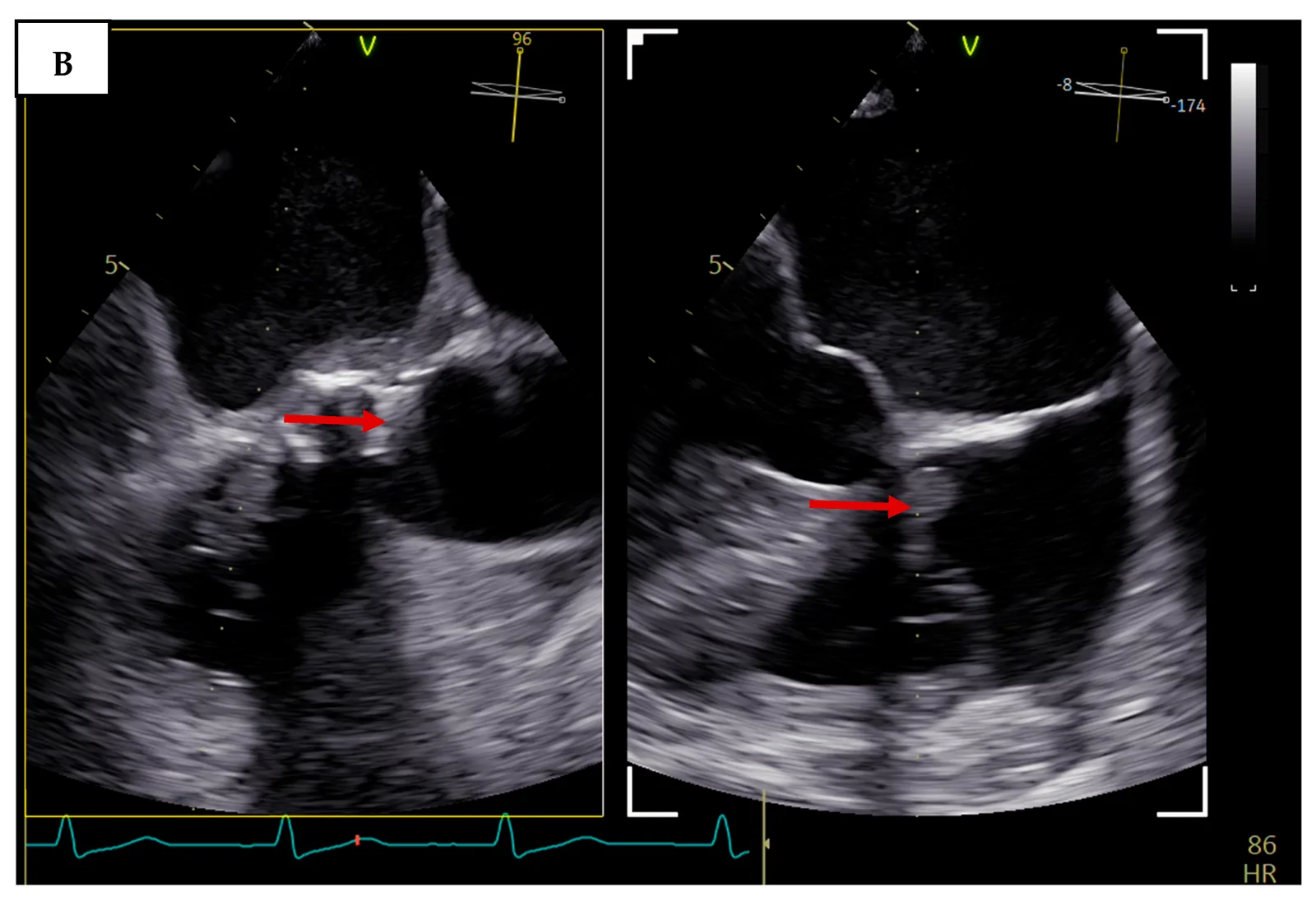
Transesophageal echocardiographic findings of persistent TAVI-associated prosthetic valve endocarditis. Mid-esophageal transesophageal echocardiographic views demonstrate residual vegetative material attached to the transcatheter aortic valve prosthesis (red arrows). (**A**) A heterogeneous echogenic periprosthetic infiltrative area (green arrow) is additionally demonstrated, raising suspicion of persistent local/periprosthetic involvement despite prolonged antimicrobial therapy. (**B**) Residual vegetative material attached to the transcatheter aortic valve prosthesis is demonstrated from additional mid-esophageal transesophageal echocardiographic views (red arrows).

**Figure 2:**
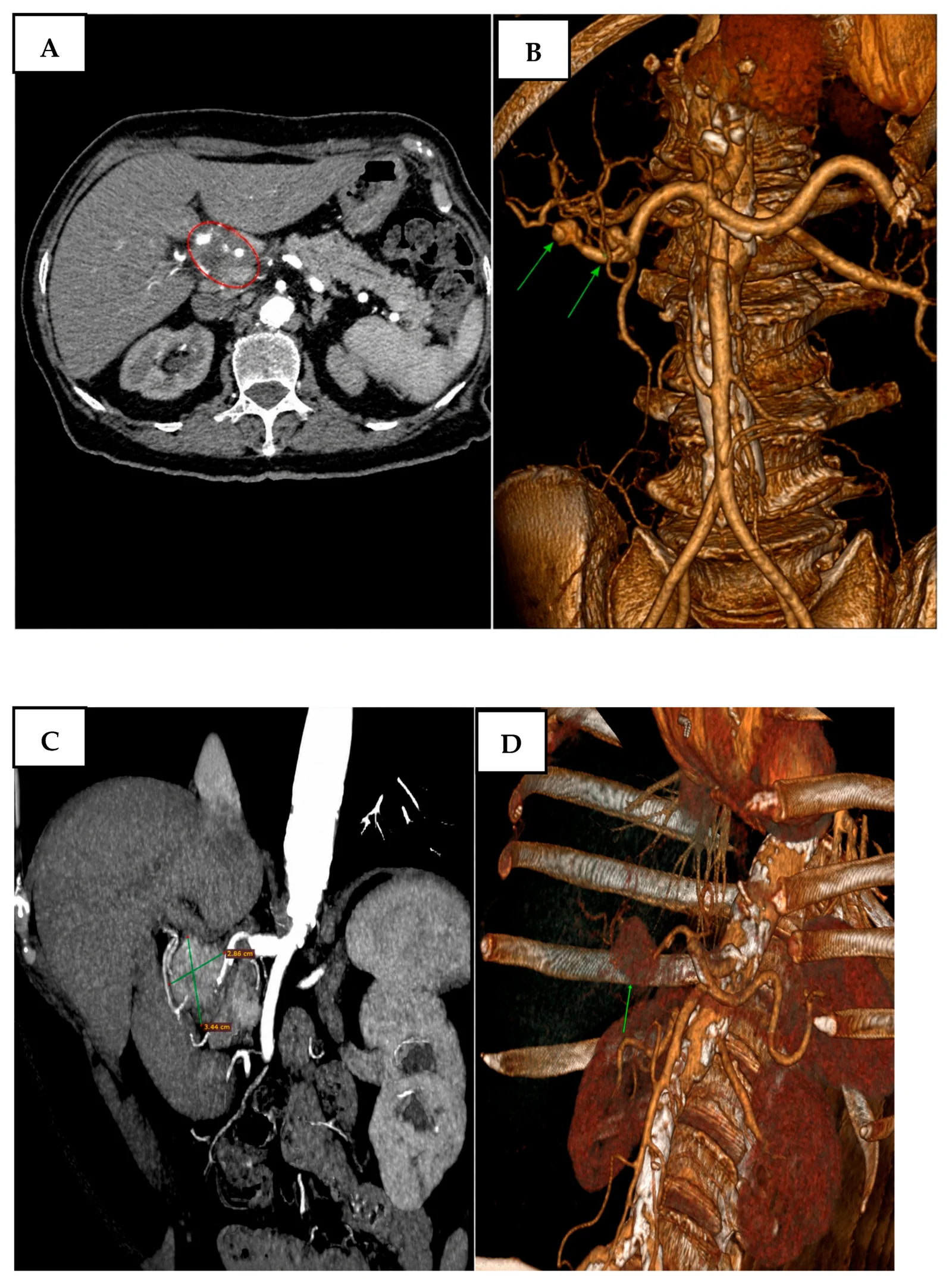
Initial and follow-up CT imaging of hepatic artery mycotic aneurysms. Upper row: (**A**) Initial axial contrast-enhanced CT demonstrating the hepatic arterial aneurysmal lesions with surrounding inflammatory changes; (**B**) corresponding three-dimensional CT angiographic reconstruction demonstrating the two focal aneurysmal lesions (green arrows). Lower row: (**C**) Follow-up contrast-enhanced CT demonstrating a single enlarged aneurysmal formation at the site of the previously identified lesions; (**D**) corresponding three-dimensional CT angiographic reconstruction demonstrating the relationship of the enlarged aneurysmal formation to the hepatic arterial circulation. Follow-up imaging demonstrated marked interval anatomical progression despite clinical and inflammatory improvement.

**Figure 3:**
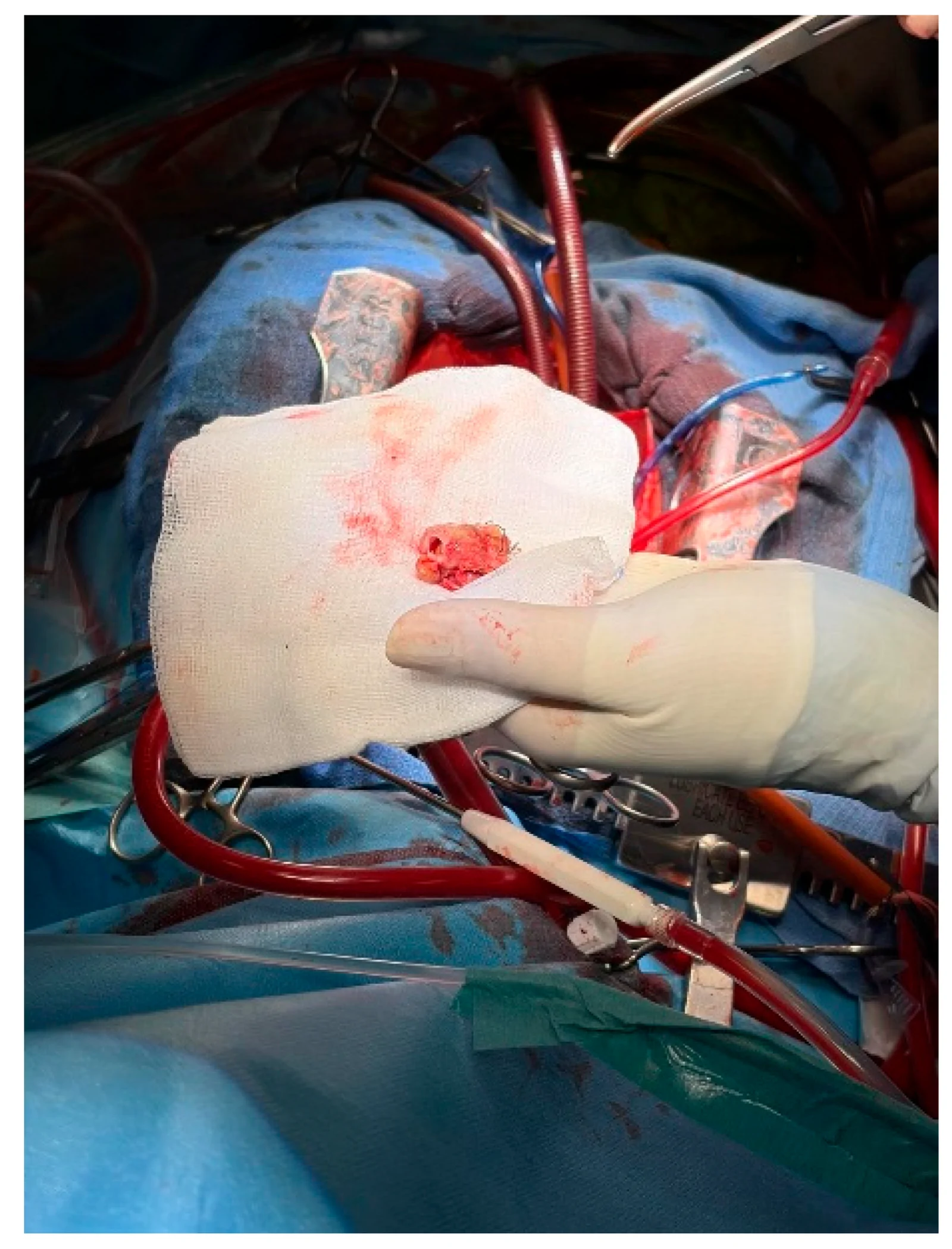
Intraoperative specimen obtained during redo surgical aortic valve replacement. The photograph shows prosthetic-associated inflammatory and vegetative tissue excised during explantation of the transcatheter aortic valve and radical debridement of the affected aortic annulus.

**Figure 4:**
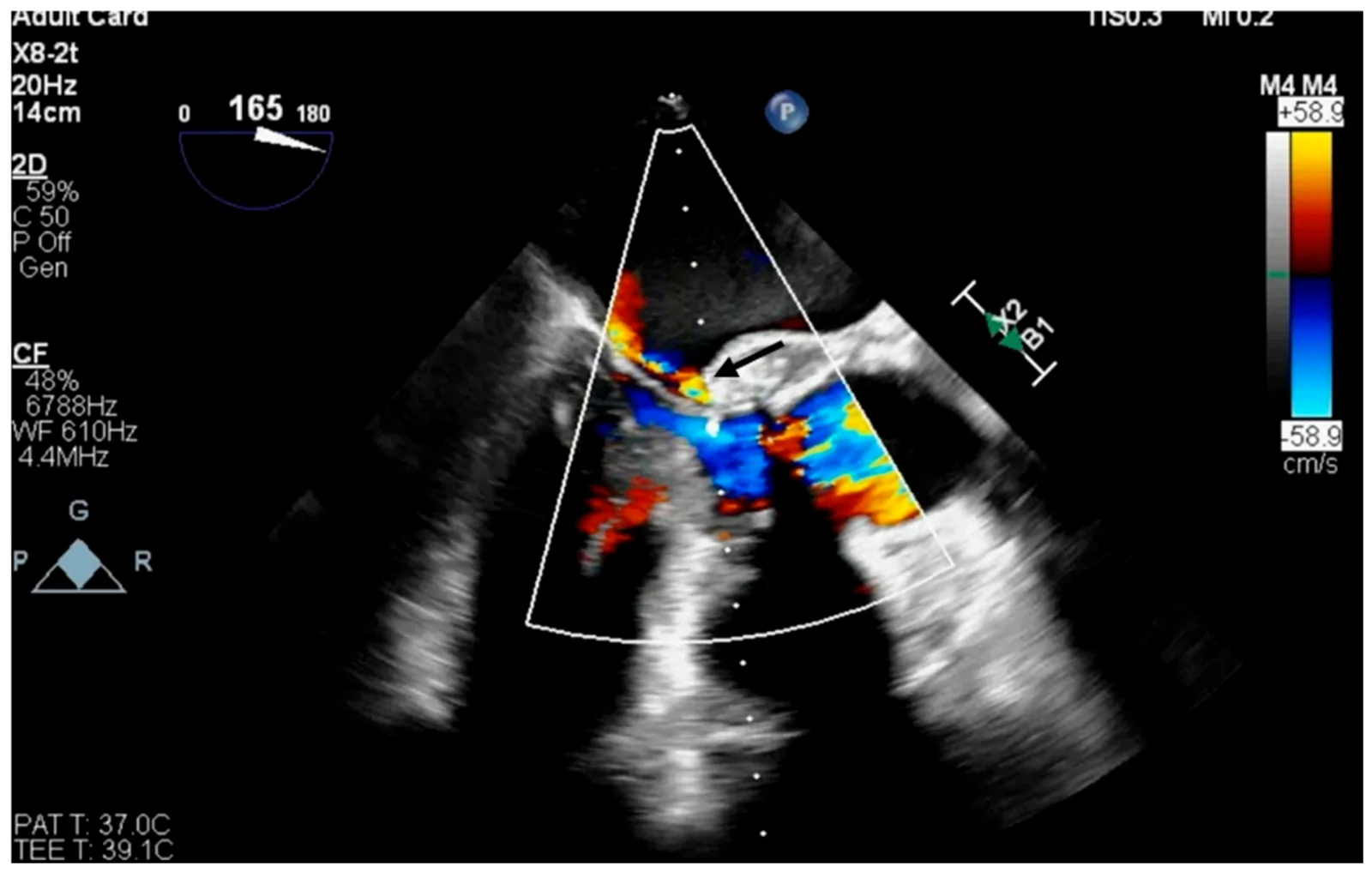
LVOT–left atrial fistulous communication identified after redo aortic valve surgery. Transesophageal color Doppler echocardiography demonstrates a small fistulous communication between the left ventricular outflow tract and the left atrium (black arrow). The communication was not identified on the available preoperative TEE examinations or during intraoperative inspection and was considered hemodynamically insignificant on postoperative assessment.

**Figure 5:**
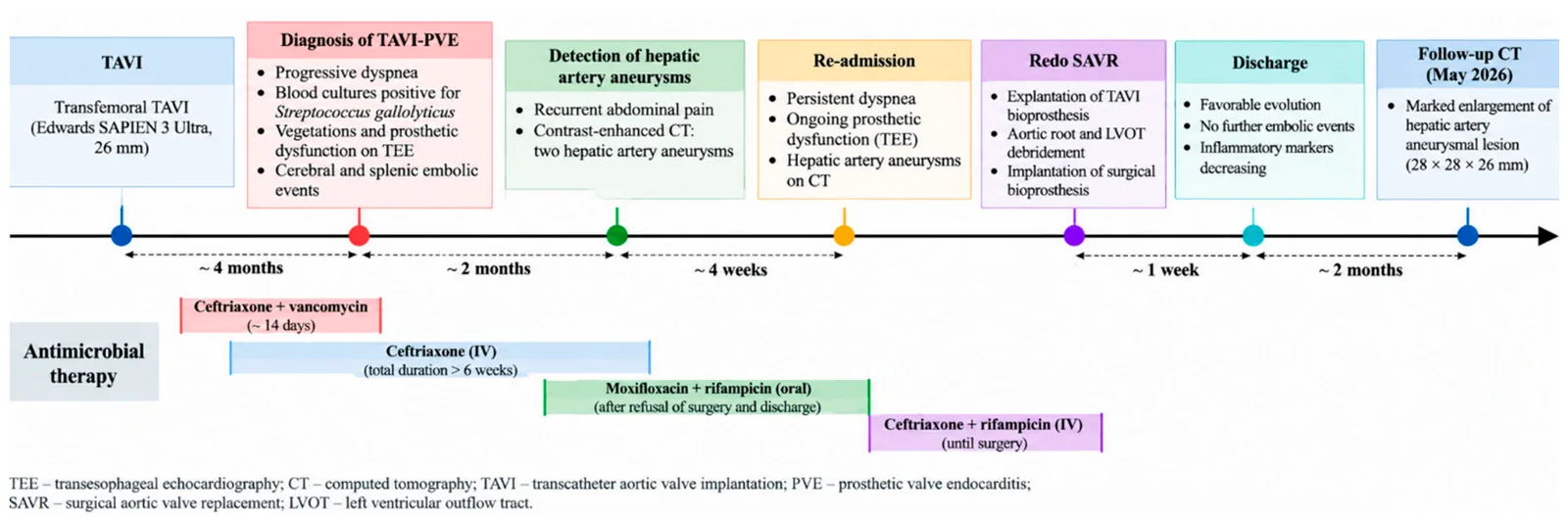
Timeline of the clinical course, antimicrobial treatment, major complications, imaging findings, and interventions.

## Data Availability

The data supporting the findings of this study are available from the corresponding author upon reasonable request. The data are not publicly available due to privacy and ethical restrictions.
